# Visualization of Cell Membrane Tension Regulated by the Microfilaments as a “Shock Absorber” in Micropatterned Cells

**DOI:** 10.3390/biology12060889

**Published:** 2023-06-20

**Authors:** Xianmeng Wang, Na Li, Zhengyao Zhang, Kairong Qin, Hangyu Zhang, Shuai Shao, Bo Liu

**Affiliations:** 1 School of Biomedical Engineering, Faculty of Medicine, Dalian University of Technology, Dalian 116024, China; 2 Liaoning Key Laboratory of Integrated Circuit and Biomedical Electronic System, Dalian University of Technology, Dalian 116024, China; 3 School of Life and Pharmaceutical Sciences, Dalian University of Technology, Panjin 124221, China

**Keywords:** macropattern, FRET, cell membrane tension, microfilament, focal adhesion

## Abstract

**Simple Summary:**

The mechanical cues in the extracellular cell matrix (ECM) regulate multiple cell behaviors by dominating the membrane tension through the cell membrane–cytoskeleton–focal adhesions (FAs) complex. However, the mechanism still needs clarification, due to the lack of technical means to alter the cytoskeleton arrangement and FAs distribution artificially. This study utilizes polydimethylsiloxane stamps with specific shapes to solve the issue and quantifies the order degree of cytoskeleton and membrane tension by defining the concept of information entropy. The results showed that the actin filaments arrangement and FAs distribution in the cells patterned by stamps were changed significantly and further regulated the membrane tension. The actin filaments accumulated in the zone where FAs were rare to maintain the stability of the overall membrane tension. In this process, the actin filaments act as shock absorbers to cushion the alternation in membrane tension without changing the steady state plasma membrane tension. Generally, this study offers an effective tool for the in-depth analysis of cell shape and function and a new method for the artificial regulation of cell function changes.

**Abstract:**

The extracellular stress signal transmits along the cell membrane–cytoskeleton–focal adhesions (FAs) complex, regulating the cell function through membrane tension. However, the mechanism of the complex regulating membrane tension is still unclear. This study designed polydimethylsiloxane stamps with specific shapes to change the actin filaments’ arrangement and FAs’ distribution artificially in live cells, visualized the membrane tension in real time, and introduced the concept of information entropy to describe the order degree of the actin filaments and plasma membrane tension. The results showed that the actin filaments’ arrangement and FAs’ distribution in the patterned cells were changed significantly. The hypertonic solution resulted in the plasma membrane tension of the pattern cell changing more evenly and slowly in the zone rich in cytoskeletal filaments than in the zone lacking filaments. In addition, the membrane tension changed less in the adhesive area than in the non-adhesive area when destroying the cytoskeletal microfilaments. This suggested that patterned cells accumulated more actin filaments in the zone where FAs were difficult to generate to maintain the stability of the overall membrane tension. The actin filaments act as shock absorbers to cushion the alternation in membrane tension without changing the final value of membrane tension.

## 1. Introduction

All cells endure complex three-dimensional mechanical microenvironment stimuli such as shear stress, tensile stress, and positive stress. The mechanical force sensed by cells affects the activity of some proteins and gene expression, and further changes the morphology and function of cells. Thus, these forces take place in connection with the occurrence and development of malignant tumors, atherosclerosis, and other diseases [1]. For example, when pressure is applied to human nasal epithelial cells, the cytoskeleton increases the beating speed of cilia and prevents mucus stasis by reshaping in the early stage of airway obstruction [2]. External matrix stiffness drives epithelial–mesenchymal transformation and tumor metastasis through the TWIST1-G3BP2 mechanical transduction pathway [3]. Therefore, it is of great significance to explore how external mechanical stimuli act on cells and how cells respond to external mechanical stimuli, to reveal the nature of functional changes to cells under corresponding conditions and to diagnose, evaluate, or treat those related diseases. Early studies generally hypothesized that external mechanical stress first acted on the cell membrane. This opened the plasma channels of Ca^2+^ [4] and K^+^ [5] in the cell membrane, increasing the corresponding ion concentration in the cell and then regulating the activity of related proteins. External mechanical stress can also directly act on cell membrane proteins, their conformational changes, and the activation of intracellular signal proteins that regulate gene expression and protein synthesis. Mechanical signals are transduced into intracellular biochemical signals after passing through the cell membrane. However, these signal substances diffuse uniformly within the cytoplasm during this process. Therefore, it is difficult to explain the phenomenon of directional migration and polarity formation of cells under stress [6]. There is a view that the extracellular mechanical force mediating polarized cell behaviors depends on the intracellular stress transmission along cellular physical structures.

Existing evidence suggests that mechanical stress can be transmitted along the cytoskeleton to the distal end when extracellular stress is applied to cells, thereby changing the activity of focal migration-related proteins and the cell polarity [7,8]. In this process, the stress signal first causes the disturbance in the membrane lipid raft and the change in membrane fluidity, leading to the change in membrane tension. Stress changes continue to be transmitted along the cytoskeleton to focal adhesions (FAs) to initiate a series of intracellular signal chains that transduce mechanical stress signals into biochemical signals [9].

In the above process, cell membrane, cytoskeleton, and FAs form a functional complex that plays the role of stress transducer. Cell membrane tension, as an actual physical property of the cell membrane, can regulate many cellular behaviors, such as cell polarization [10], cell migration [11], cell diffusion [12], and cell membrane repair [13]. Exploring the tension changes in cell membranes contributes to understanding the intracellular force transmission and polarity establishment. However, due to the shortcomings of existing methods to measure membrane tension, the nature of the membrane tension change in response to external stress still needs to be clarified. The cytoskeleton can directly mediate stress variation in the membrane. The physical transfer of stress along the cytoskeleton may also affect the reconstruction of the cytoskeleton itself [14]. However, since it is not easy to alter the arrangement and distribution of the cytoskeleton artificially, whether the morphology, arrangement, and quantity of the cytoskeleton affect this signaling process still needs to be determined. Stress can also alter the distribution of FAs [15], affect the arrangement of the cytoskeleton, and further regulate cell membrane tension. For example, the tension in FAs regulates the clathrin- and dynamin-independent endocytic pathway (CLIC/GEEC pathway) through vinculin and, ultimately, affects membrane tension [16]. When podocytes are inoculated on the uncoated substrate, destruction of the actin cytoskeleton is observed due to FAs damage, along with the generation of membrane vesicles [17]. Nevertheless, due to the lack of technical means to change the distribution of FAs, further study is still needed to reveal its regulatory mode and the specific mechanism.

Based on these issues, this study designed micropatterned seals for culturing patterned cells to alter the cytoskeleton arrangement and FAs’ distribution artificially. The membrane tension of the patterned cells was investigated by a DNA-encoded fluorescence resonance energy transfer (FRET) biosensor with high temporal resolution under static and dynamic conditions to explore its role in the process of stress transfer. These results showed that, when micropatterned cells were stimulated with a hypertonic solution, the arrangement of cytoskeletal filaments directly affected the change in membrane tension, the density of cytoskeletal filaments reduced the magnitude of the change in membrane tension, and the absence of focal adhesions exacerbated the magnitude of changes in membrane tension.

## 2. Materials and Methods

### 2.1. Preparation of Substrate with Micropatterns

**Figure 1:**
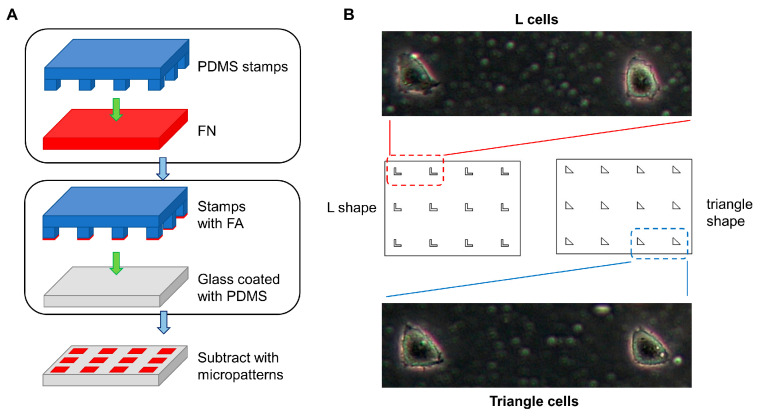
Culturing patterned cells on micropatterns. (**A**) The flow diagram used to prepare the substrate with micropatterns. (**B**) The micropattern matrix and the patterned cells.

The L shape and triangle micropattern size was 20 μm × 20 μm. The micropatterns were arranged in a 3 × 4 matrix and engraved on a silicon template. The liquid polydimethylsiloxane polymer (PDMS, Midland, TX, USA) was mixed with the curing agent (Sylgard 184, Midland, TX, USA) in a 10:1 volume ratio and stirred with a glass stirring rod for about 10 min, until the mixture was uniform. The mixture was poured over the silicon template, engraved with micropatterns, placed on the heating plate at 85 °C for 90 min, and then cooled for 5 min to obtain the PDMS stamps. The clean cover glass covered with the same polymer mixture was rotated in the homogenizer at a speed of 3400 RPM for 1 min to ensure a uniform PDMS substrate. The stamps were incubated with fibronectin solution (FN, 0.1 mg/mL, 25 μL, Sigma, St. Louis, MO, USA) for 1 h. After the solution dried, the stamps were pressed on the PDMS substrate for 12 h to obtain a substrate with micropatterns (Figure 1A).

### 2.2. Culture of Patterned Cells and Plasmids

Chinese hamster ovary cells (ATCC) were cultured in Ham’s F 12 nutrient medium (Hyclone, Logan, UT, USA), containing 10% FBS (Bioind, Kibbutz Beit Haemek, Israel), 100 unit/mL penicillin, and 100 μg/mL streptomycin (Hyclone, Logan, UT, USA), in a humidified incubator with an atmosphere of 95% O_2_ and 5% CO_2_ at 37 °C. Cells were seeded on the substrate with micropatterns and cultured for 4 h before observation. The DNA plasmids used in this work contained PaxTS (Dalian, China) to visualize the tension across paxillin and the MSS biosensor to measure the cell membrane tension. Both the DNA plasmids were transfected into cells by Lipofectamine 3000 reagent (Invitrogen, Carlsbad, CA, USA) according to the company protocol.

### 2.3. The Cytoskeletal Filamemts Staining and FAs Localization

The cells were washed twice with PBS (CellPro, Suzhou, China), fixed for 10 min with 4% paraformaldehyde, washed again with PBS, incubated in phalloidin (Macklin, Shanghai, China) in the dark for 90 min, and, finally, washed twice with PBS. The PaxTS, which detects the tension across paxillin, was applied to localize the FAs.

### 2.4. Microscope Image Acquisition

The microscope image acquisition set-up contained an inverted microscope (Olympus, IX73, Tokyo, Japan), a 420DF20 excitation filter, a 455DRLP dichroic mirror, and two emission filters controlled by a filter controller (480DF30 for CFP and 535DF25 for YFP). All fluorescence images were collected on a single isolated cell by Software (Cellsens Dimension 1.18) with an interval of 1 min at 60 magnifications.

### 2.5. Image Snalysis and Statistical Analysis

The FAs were identified by the method proposed in previous work [18]. The FRET index is the FRET ratio between the Ypet and enhanced cyan fluorescent protein (ECFP) channels. The ‘One-way ANOVA’ method in ‘Column analyses’ in GraphPad Prism 9 software was used for statistical analysis to evaluate the statistical difference between groups. A significant difference was determined by the *p*-value (<0.05). The data population numbers (n) were the number of cell samples in each group.

### 2.6. Calculation of Angular Information Entropy of Cytoskeleton and Membrane Tension Information Entropy

Fluorescence images of cytoskeleton microfilaments were imported into the Image pro plus software. The microfilaments were marked, and the angles between the microfilaments and the vertical axis were calculated. The angles between microfilaments and the vertical axis (0–90°) were divided into six parts, 15° for each, and their frequency in each part was represented by P_i_ (P_1_, P_2_, P_3_, P_4_, P_5_, and P_6_). The angle information entropy (*AIE*) was calculated by Formula (1):

(1) $$AIE=-\sum_{1}^{6}P_{i}{log}_{6}P_{i}$$

Membrane tension fluorescence images of “L” cells were imported into the Image pro plus software. Each cell was divided into 50 parts along the direction of cytoskeleton filaments; then, each part of the value was calculated, and the value (0–2) was divided into 10 parts, 0.2 for each—their frequency in each part was represented by P_i_ (P_1_, P_2_, P_3_, P_4_, P_5_, P_6_, P_7_, P_8_, P_9_, and P_10_). The membrane tension information entropy (*MTIE*) was calculated by Formula (2):

(2) $$MTIE=-\sum_{1}^{10}P_{i}{log}_{10}P_{i}$$

## 3. Results

### 3.1. Cell Patterning Alters the Arrangement of Cytoskeletal Filaments and the Distribution of FAs

**Figure 2:**
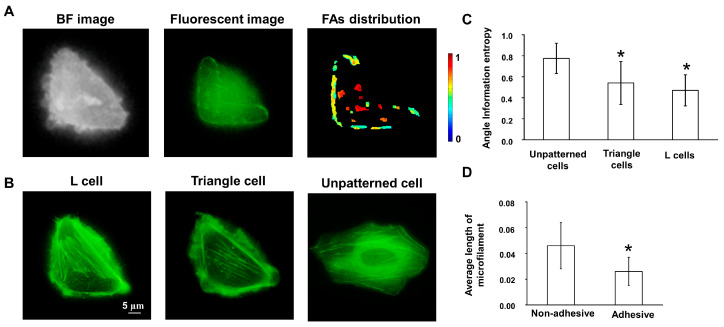
The properties of patterned cells were changed. (**A**) FAs only assembled on the micropatterns. The images present bright field image, fluorescent image from the FRET channel, and FAs’ distribution on L cells from left to right, respectively. (**B**) The staining images of cytoskeletal actin. (**C**) The average length of microfilaments in the patterned and unpatterned zones. * indicated a statistical significance compared with the unpatterned cells. (**D**) Angle information entropy of unpatterned cells, triangle cells, and L cells. * indicated a statistical significance compared with non-adhesive zone.

The substrate with micropatterns is shown in Figure 1B. The cells seeded on micropatterns were patterned cells that contained the L cells cultured on L shape micropatterns and triangular cells on triangular micropatterns (Figure 1A). L and triangle cells exhibited specific shapes compared to those growing on the substrate without micropatterns. However, L cells spread out of the micropatterns, eventually forming a triangle shape. The PaxTS biosensor marked the FAs’ location, showing that cells grew on the micropatterns, as expected. However, FAs of L cells only assembled on the L shape micropatterns (Figure 2A). Thus, the L cells were considered in two zones: the adhesive zone, which was the inner of the two micropatterns, and the non-adhesive zone, where the body of L cells was outside the micropatterns. This suggested that the micropatterns successfully controlled the shape and FAs distribution of cells.

The cytoskeletal filaments’ stain displayed that the filamentous stress fibers in the triangular cells was mainly aligned along the side of the micropatterns and throughout the cell body (Figure 2B). The filamentous stress fibers aligned in both the adhesive and non-adhesive zones of L cells along the diagonal of L micropatterns. However, the average length of filamentous fibers in the non-adhesive zone was larger than in the adhesive zone, and the filamentous fiber was denser in the non-adhesive zone than in the adhesive zone (Figure 2C). To further describe the arrangement of microfilaments quantitatively, the value of *AIE* was introduced, which implied a more disordered filamentous stress fiber network with a higher value. The results showed that the *AIE* values of triangle cells and L cells were 0.60 ± 0.20 (n = 42) and 0.47 ± 0.15 (n = 40), respectively, which were significantly lower than unpatterned cells (0.78 ± 0.14, n = 41, *p* < 0.05, Figure 2D). This demonstrated that the micropatterns altered the arrangement of cytoskeletal filaments artificially, and the stress fiber assembled more in the zone where the cell body did not adhere to the substrate.

### 3.2. Cell Membrane Tension Is Independent of Cytoskeleton and FAs’ Distribution under Static Conditions

**Figure 3:**
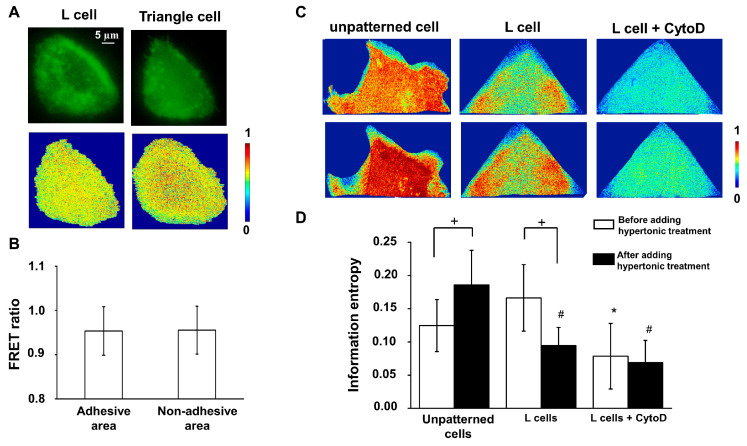
The cell membrane tension before and after the hypertonic treatment. (**A**) The cell membrane tension fluorescence image (FRET channel) and the FRET ratio image. (**B**) The histogram of FRET ratio of the adhesive and non-adhesive zones. (**C**) The FRET images showing the cell membrane tension changes upon the hypertonic treatment. The top row is before the treatment, and the below is after. (**D**) The histogram of MITE under different conditions. * Indicates a statistical significance compared with normal cells before the addition of stimulus (*p* < 0.05), # indicates a statistical significance compared with normal cells after the addition of stimulus (*p* < 0.05), + indicates a statistical significance before and after the addition of stimulus.

The MSS biosensor linked the lipid rafts to the regions without lipid rafts by a tension sensor module which consisted of a pair of FRET proteins and a nano-spring, measuring the cell membrane tension by the FRET index. This work considered the FRET ratio as the FRET index of the MSS biosensor to evaluate the impact of micropatterns on the cell membrane tension. The increase in membrane tension stretched the nano-spring and led to a FRET ratio decrease. Here, we divided an L cell into 50 parts along the diagonal of the micropatterns, which was the main arrangement direction of filamentous stress fibers, and calculated the ratio value of each part, as well as the *MTIE*. The calculation indicated no local difference in the FRET ratio along the direction of the cytoskeletal filaments’ arrangement. In addition, the *MTIE* value of L cells (0.17 ± 0.05, n = 20) was similar to the unpatterned cells (0.12 ± 0.04, n = 25, *p* > 0.05). A higher *MTIE* value referred to the membrane tension distributed more inhomogeneously. Hence, the results found that the distribution of membrane tension in triangular cells and L cells was homogeneous under static conditions (Figure 3A,B). Furthermore, there was no FRET ratio difference between the adhesive and non-adhesive zones (*p* > 0.05, n = 36, Figure 3B). Generally, this implied that the distribution of membrane tension under a static state was independent of the FAs’ location and filamentous actin fibers’ arrangement.

### 3.3. The Arrangement of Cytoskeletal Filaments Directly Affects the Changing Pattern of Membrane Tension

Considering the connection between the plasma membrane and cytoskeleton, the stress fibers network was still likely to participate in the cell membrane’s resistance to extracellular stimulus. Thus, we further utilized sucrose solution to apply hypertonic treatment (0.05 g/mL) to the cells to estimate the role of filamentous fibers’ arrangement in regulating the cell membrane tension. The result indicated that the *MTIE* value of normal cells after hypertonic treatment increased to 0.19 ± 0.05 (n = 25), higher than that before (*p* < 0.05). However, the *MTIE* value of L cells decreased to 0.09 ± 0.03 (n = 20), lower than that before adding stimulation (*p* < 0.05), as shown in Figure 3C. The hypertonic treatment resulted in the *MTIE* value of L cells being significantly lower than that of unpatterned cells (*p* < 0.05, Figure 3D).

**Figure 4:**
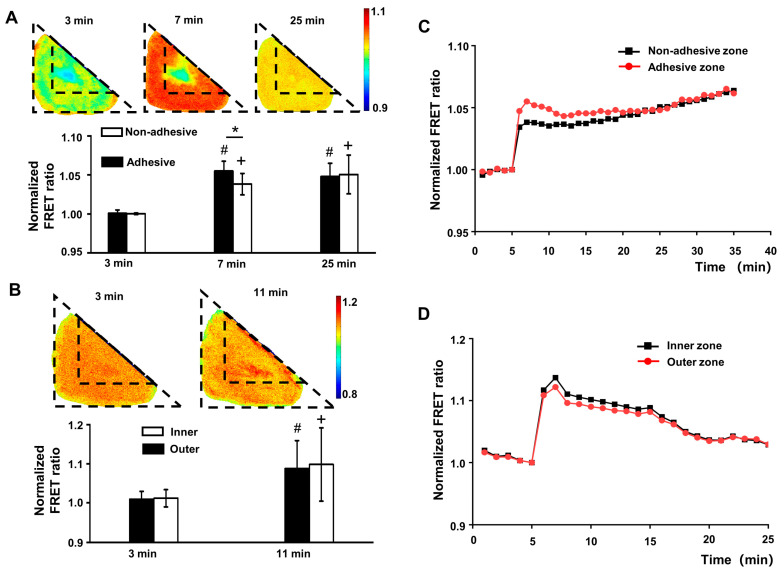
The membrane surface tension in different cells changed differently after the hypertonic treatment. (**A**) The FRET images of membrane surface tension of L cells and the histogram showing the FRET ratio of the adhesive and non-adhesive zones at specific time points. * indicates a statistical significance between the two zones (*p* < 0.05), # and + indicate a statistical significance compared with before the hypertonic treatment (*p* < 0.05). (**B**) The FRET images of membrane surface tension in triangle cells and the histogram showing the FRET ratio of the inner and outer zones at specific time points. # and + indicate a statistical significance compared with before the addition of hypertonic treatment (*p* < 0.05). (**C**) The normalized time series showing the FRET ratio changes in the adhesive and non-adhesive zones of L cells. (**D**) The normalized time series showing the FRET ratio changes in the inner and outer zones of triangle cells. The treatment was applied at 6 min.

Although the hypertonic treatment increased the FRET ratio integrally, further analysis found that the ratio increase in the adhesive zone was much more significant than in the non-adhesive zone (*p* < 0.05). This difference gradually narrowed and disappeared at 20 min (Figure 4A,C). However, the inner zone of the triangular cells, which was the same location as the non-adhesive zone of L cells, showed no FRET ratio difference from the outer zone after the hypertonic treatment (*p* > 0.05, Figure 4B,D). An interesting phenomenon was that the FRET ratio of L cells increased, sharply at first and then slowly, after the hypertonic treatment, while the FRET ratio of triangle cells increased sharply and then decreased slowly to a level similar to L cells. The stress fiber arrangement differed between the adhesive and non-adhesive zones, implying that the regulation of membrane tension upon the external stimulus was associated with the arrangement of stress fiber.

**Figure 5:**
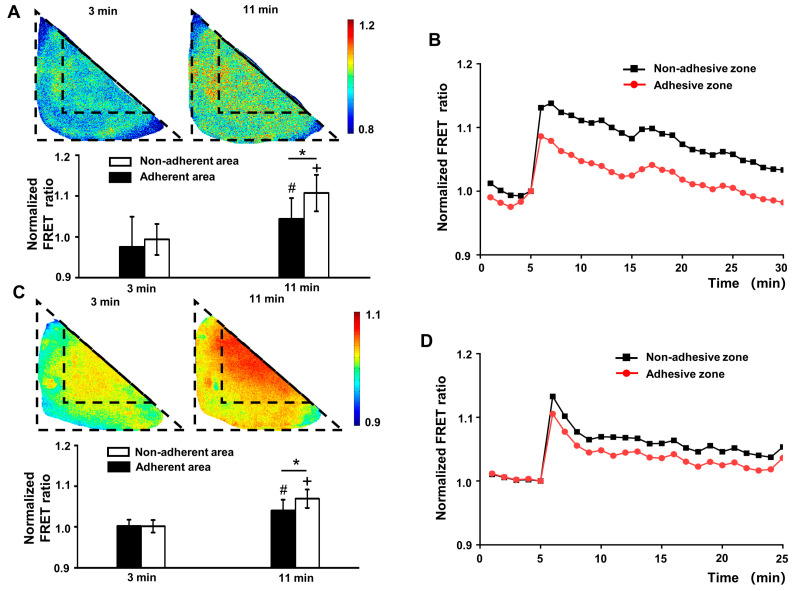
The different changes in membrane surface tension of L cells after the hypertonic treatment were related to the actin filaments and FAs. (**A**) The FRET images of membrane surface tension in L cells treated with ML-7, and the histogram showing the FRET ratio of the adhesive and non-adhesive zones at specific time points. * indicates a statistical significance between the two zones (*p* < 0.05), # and + indicate a statistical significance compared with before the hypertonic treatment (*p* < 0.05). (**B**) The normalized time series showing the FRET ratio changes in the adhesive and non-adhesive zones of L cells treated with ML-7. (**C**) The FRET images of membrane surface tension in L cells treated with CytoD (Sigma, St. Louis, MO, USA), and the histogram showing the FRET ratio of the adhesive and non-adhesive zones at specific time points. # and + indicate a statistical significance compared with before the addition of hypertonic treatment (*p* < 0.05). (**D**) The normalized time series showing the FRET ratio changes in the adhesive and non-adhesive zones of L cells treated with CytoD.

To investigate whether the mechanical properties of filaments caused this difference, the myosin light chain kinase MLCK inhibitor (ML-7, Sigma, St. Louis, MO, USA) was added to L cells to inhibit the contraction of stress fibers, followed by sucrose solution. After the hypertonic stimulation, the FRET ratio in both adhesive and non-adhesive zones increased significantly over a short time but decreased slowly after that (Figure 5A). The increase in the non-adhesive zone was higher than in the adhesive zone (*p* < 0.05). The difference persisted over the observation period of 20 min after the hypertonic stimulation (Figure 5C). This suggested that the regulation of membrane tension upon stimulus depended on the filamentous fibers, which likely cushioned the impact of external stimulus on the membrane tension.

### 3.4. The FAs Functions in Regualting the Memrbane Tension

The distribution of FAs in the adhesive and non-adhesive zone was also different. To further explore whether FAs regulated the membrane tension via the cytoskeleton, CytoD was applied to destroy the structure of L cell filaments. This revealed that depolymerizing filamentous stress fibers decreased the *MTIE* value of L cells significantly under a static state (0.08 ± 0.05, n = 23, *p* < 0.05) compared to the unpatterned cells. Interestingly, there was no *MTIE* difference between before and after the hypertonic treatment applied to the L cells treated with CytoD (Figure 3D). Meanwhile, the FRET ratio increase in the non-adhesive zone of L cells was higher than that of the adhesive zone (*p* < 0.05). The difference remained for 20 min and did not disappear (Figure 5C). This revealed that the FAs regulated the membrane tension upon external stimulus, together with the cytoskeletal filaments.

## 4. Discussion

This work utilized stamps to print triangle and L shape micropatterns on the PDMS substrate with FN. This ensured that the cells only adhered to the micropatterns via FAs, generating an adhesive zone with the feature of a triangle or L shape integrally. The cells cultured on the triangle and L shape micropatterns both showed triangle bodies and filamentous stress fibers mainly aligned along the axis of the cell bodies, displaying higher uniformity than the cells not patterned by micropatterns. The results demonstrated that culturing cells on the substrate with micropatterns printed by stamps with FN artificially controlled the FAs’ distribution and cytoskeleton arrangement. Interestingly, the *AIE* value of triangle cells was higher than L cells, probably because the triangle shape micropatterns could not pattern the orientation of actin filaments. This work observed highly ordered filaments with different orientations in triangle cells (Figure S1), but actin filaments in L cells showed high uniformity and orientation. Thus, the L cells performed better in patterning the actin filaments’ alignment. In addition, L cells provided local zones with different adhering states. The cells on L micropatterns ultimately developed into triangle shapes. However, the inner portion of L cells merely lay on the substrate without connection and generated a non-adhesive zone instead of adhering through FAs. This demonstrated that the cells assembled longer and denser stress fibers in the non-adhesive zone without FAs, implying a compensatory mechanism between the FAs and actin stress fibers.

In neutrophils, microtubule drips increase surface tension and inhibit cell front formation and signal transduction for several seconds, whereas decreasing surface tension produces the opposite effect [18]. This suggests that plasma membrane (PM) tension can also regulate intracellular signaling pathways, so the diffusion of PM tension from a non-uniform state at the beginning of adherent to the uniform state under stable conditions may also lead to cytoskeleton aggregation. Beneath the plasma membrane lies a three-dimensional (3D) cytoskeletal network with an irregular actin meshwork and highly ordered actomyosin structure. In nonmuscle cells, the most prominent actomyosin structures are stress fibers, which are thick actin bundles that are interdigitated with bipolar nonmuscle myosin II (NMII) filaments and are usually associated with FAs at their ends [19]. The mutual sliding of actin and myosin II filaments drives the contraction of stress fibers to generate force, enabling force transmission along the stress fibers [20]. The actin cytoskeleton connects to the ECM via FAs to build bidirectional and mechanosensitive communication between the cell and extracellular environment. Force transmitted from the cytoskeleton through FAs to the ECM is commonly considered the traction force necessary for rigidity sensing, cell shape, adhesion, and migration [21]. Cells only adhering to the micropatterns changed their shape and actin stress fiber arrangement accordingly. Thus, the L shape micropatterns patterned a non-adhesive zone in the center of the L cells. No FAs were inside this zone to connect the cytoskeleton to the ECM and generate enough force to maintain the cell shape. Only the FAs assembled along the inner edge of the L shape micropatterns provided connections for the stress fibers in the adhesive zone. Based on the limited FAs, the adhesive zone gathers longer and denser stress fibers [22]. The mechanical properties of individual actin filaments depend on their length [23]. The number of NMII molecules assembled to an actin filament is limited. Hence, the force generation of a single stress fiber is limited [22]. The cell organizes more stress fibers in the adhesive zone, signifying an increase in the local scale of force generation to supplement the force that FAs are unable to provide to maintain the cell shape. Generally, FAs and stress fibers seem to compose a synergic structure and are together responsible for the mechanical steady state of cells, which could also be understood as a compensatory mechanism of the cell itself. When FAs are hard to assemble and grow, cells recruit more stress fibers to provide mechanical support to maintain cell shape and even out the stability.

The stability discussed in this work is mainly about the PM tension, which was considered to be the relative motion between lipid rafts and the zone outside raft domains on the plasma membrane. Without mechanical load, the PM tension of L cells was uniform globally, and its uniformity was similar to the unpatterned cells, suggesting the final steady state of PM tension was unrelated to the locations of FAs and arrangements of actin filaments. However, these two factors seemed to regulate how PM tension responded to external hypertonic treatment. The high orderliness of stress fibers led to the external mechanical load induced-PM tension changing more uniformly, supporting that the regulation by cytoskeleton of the PM tension depended on the arrangement of filamentous actin. Several cytoskeleton proteins interact with lipid rafts to form raft–cytoskeleton binding protein complexes that control cell migration and adhesion [24]. The physical connection controls the range of raft motion. The longer and denser stress fibers provide more force to anchor the rafts to resist the mechanical load, and the high orderliness causes the raft motions to be similar upon external mechanical disturbance. Thus, the distribution of PM tension is more uniform. When CytoD destroyed the cytoskeleton microfilament structure, the MITE value reached the minimum, suggesting a more uniform distribution of PM tension. This can be understood to show that the plasma membrane is not supported by microfilaments. The PM tension cannot transmit to the cytoskeletal actin through protein. Without the cytoskeleton, the responsiveness of cell contraction to chemical stimulation reduces [25], and a significantly different contractile behavior emerges [18]. Therefore, the interference of actin filaments to spread the tension disappeared, which can be approximately considered as a uniform diffusion process. In unpatterned cells, the ability of stress fibers to anchor rafts is weaker due to the length and density of actin filaments, and, hence, the rafts swing largely when disturbed by mechanical load. The fibers are also disoriented, resulting in the motions of lipid rafts being random. The above conclusion is also proven from the side force inside cells, by the fact that, from the cell membrane to the cytoskeleton, actin filaments participated in the process mainly through the role of the protein directly connect to the cell membrane, contributing to the spread of mediated tension.

The claim that the arrangement of cytoskeletal actin regulates the PM tension is also supported by the value changes in PM tension upon mechanical load. The PM tension decreased suddenly after hypertonic treatment, illustrated by a FRET ratio leap, due to the cell shrinkage. Nevertheless, the PM tension in triangle cells performed more sensitively to the external stimulus, with a more significant reduction than in L cells. Then, the triangle cells experienced a slow increase in PM tension, but the PM tension in L cells kept a slow decrease. Finally, the PM tension in the triangle and L cells met similarly. It has been reported that cells respond to external mechanical perturbations elastically at short time scales [26], and the cytoskeleton buffers external mechanical force application [27]. The performance of triangle and L cells conformed with this mechanical disturbance-induced elastic response, cushioning the impact from the external mechanical load, while the different modes of cushion implied the different roles of FAs and actin filaments. The other findings from L cells in this work further support the assumption that FAs and stress fibers function differently upon mechanical load. For instance, the rapid response of PM tension at the adhesive zone with more FAs and fewer stress fibers was more obvious than that at the non-adhesive zone upon hypertonic treatment.

This tension on the plasma membrane is related primarily to the connection between the membrane and ECM and its internal cytoskeletal networks [28]. Measuring the mechanical coupling between two cell membrane tethers at distances of 5–15 μm exhibits no long-distance propagation of the cell membrane at a short scale, probably due to the membrane flow disturbance being mediated by the interaction between transmembrane proteins and the underlying cytoskeleton [29,30]. This is a direct physical regulation pathway, involving cell type, cortical thickness, and cortical structure [31,32], suggesting that the cytoskeleton directly mediates membrane tension through its interaction with the membrane. Although there also exist biochemical pathways that regulate the membrane tension, the cytoskeletal components, including vinculin, spectrin, microtubules, class I and II myosins, and MCA proteins, regulate the membrane tension [33,34,35,36,37,38,39], further revealing an additional occupation of the actin filaments’ arrangement: to regulate the membrane tension physically via interaction with the membrane protein. This regulation is closely associated with the arrangement of filamentous actin. An ordered architecture is beneficial for filamentous actin to generate force, proving that stress fibers are arranged more orderly to provide more traction force for cells to adhere to the stiff subtract [40]. The actin bundles more parallel to the cell edge decrease the elasticity of the lamellipodia [41]. This implies that the cushion of the cytoskeleton also contains the ordered stress fibers which resist the mechanical load, in contrast to the traditional view that the disoriented actin filament network disperses the load [27]. Instead, the cushion does not counteract the external mechanical but slows the process to the final state.

This assumption was further proven by the results of inhibiting the contraction of actin by ML-7. The PM tension decreased sharply and increased slowly when inhibiting the contraction of cytoskeletal filaments. The force generation along fiber stress decreased due to the suppression of filament contraction. Thus, the resistance of stress fibers to the external mechanical load was limited, increasing the transient change amplitude. The subsequent slow increase was similar to triangle cells without any drug treatments in shape. This further suggests the actin filaments’ arrangements and the force transmission mode along the ordered filaments determine how PM tension returns to the steady state. However, the PM tension still functions differently in change amplitude between adherent and non-adherent zones, possibly due to the FAs.

Depolymerizing the actin cytoskeletal filaments with CytoD did not affect the FAs. The PM tension decreased sharply and then increased rapidly to a steady level until the observation ended. The tension level of the non-adhesive zone was lower than the adherent zone where the FAs were more numerous, indicating that FAs in the adhesive zone maintained the stability of PM tension after the depolymerization of microfilaments. Therefore, the change in PM tension in the non-adhesive zone is more prominent, suggesting that the loss of FAs intensifies the evolution of membrane tension. Furthermore, how FAs keep PM tension stable still needs to be clarified. The pathway is possibly involved in some biochemical signals. Previous experiments have shown that pulling the integrin on the FAs can activate the early signaling protein FAK/Src complex in FAs and initiate a series of intracellular signaling chains. Pulling the matrix on the basal surface of cells can open the calcium channel on the cell membrane and release the intracellular calcium pool, increasing intracellular calcium ion concentration. However, when the integrity of the microfilaments is destroyed, local calcium signal enhancement from the intracellular calcium pool near the basal pulling site is still observed [42]. This implies mechanical signals can be converted directly into chemical signals via FAs. Therefore, the membrane tension in the adhesive zone is transferred to FAs with less variation than in the non-adherent regions without FAs. In other words, when the cytoskeleton cannot mediate membrane tension, membrane tension is directly transformed into biochemical signals locally through FAs. The PM tension does not stabilize quickly due to the destruction of cytoskeleton filaments, which also indicates that the cytoskeleton plays a crucial role in maintaining the stability of the whole-cell membrane tension.

## 5. Conclusions

This study proposed a manner to analyze the dynamic relationship between FAs, the cytoskeleton, and PM tension quantitatively. It contained micropattern stamps to culture patterned cells, an MSS biosensor to visualize the membrane tension, and the concept of information entropy to quantify the orderliness of cytoskeleton and membrane tension. The patterned cell offered a live-cell model in which the actin filaments’ arrangement and FAs’ distribution were highly ordered. The model excluded the interference caused by the disorientated arrangement of the cytoskeleton and random distribution of FAs, which is valuable for some studies, such as studies focusing on intracellular force transmission. In this manner, this study found a compensatory mechanism between FAs and actin filaments, namely, that cells maintain the stability of PM tension via accumulating more actin filaments when lacking FAs. Upon mechanical disturbance, the ordered actin filaments resist the external load, functioning as shock absorbers to slow the impact of mechanical load on the PM tension. However, they are unable to determine the steady state of PM tension. FAs also play roles in maintaining PM tension, possibly via a biochemical pathway.

## Data Availability

The data presented in this study are available on request from the corresponding author. The data are not publicly available due to the images with PaxTS were further analyzed in another preparing work.

## Supplementary Materials

The following supporting information can be downloaded at: https://www.mdpi.com/article/10.3390/biology12060889/s1, Figure S1: The staining images of cytoskeletal actin in triangle cells.
