# Supplementary figures and images for: Visualization of Cell Membrane Tension Regulated by the Microfilaments as a “Shock Absorber” in Micropatterned Cells
Source: Biology (Basel). 2023 Jun 20;12(6):889. doi: 10.3390/biology12060889 (PMC10295218; doi:10.3390/biology12060889)

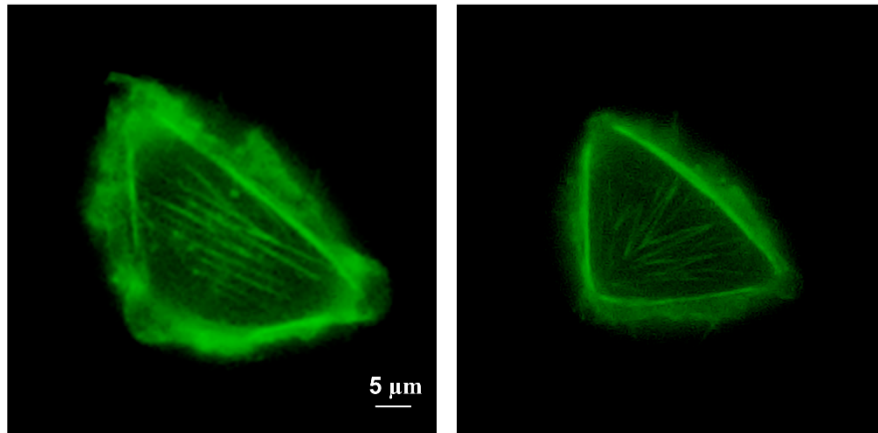

Figure S1. The staining images of cytoskeletal actin in triangle cells.

Supplement: Supplementary file 1 [file biology-12-00889-s001.zip › supplementary materials.pdf]
